# Influence of body mass index and polycystic ovarian syndrome on ICSI/IVF treatment outcomes: A study conducted in Pakistani women

**Published:** 2018-08

**Authors:** Rehana Rehman, Mohsin Mehmood, Rabiya Ali, Saeeda Shaharyar, Faiza Alam

**Affiliations:** 1 *Department of Biological and Biomedical Sciences, Aga Khan University, Karachi, Pakistan.*; 2 *Liaquat University of Medical and Health Sciences, Jamshoro, Pakistan.*; 3 *Department of Physiology, Bahria University Medical and Dental College, Bahria University medical and Dental college (BUMDC), Karachi campus, Defense Phase II, adjacent PNS Shifa Hospital.*

**Keywords:** Quetelet’s index, Obesity, Polycystic ovary syndrome, Sperm injection, Intracytoplasmic, Infertility

## Abstract

**Background:**

Obesity may establish a crucial barrier for effective fertility treatment in polycystic ovary syndrome (PCOS) females.

**Objective:**

To compare results of intra-cytoplasmic sperm injection (ICSI) in females with and without polycystic ovarian syndrome and further appraise the effect of obesity in PCOS females.

**Materials and Methods:**

A cross-sectional study from June 2015 to July 2016 included non-PCOS and PCOS (recognized by Rotterdam criteria) females who underwent ICSI. The PCOS were further stratified into non-obese and Obese according to the South Asian criteria for body mass index. Results were categorized on the basis of beta-human chorionic gonadotropin (β-hCG) and transvaginal scan into non-pregnant (β-hCG <25 mIU/ml), preclinical abortion (β-hCG >25 mIU/ml with no fetal cardiac activity) and clinical pregnancy (β-hCG >25 mIU/ml with fetal cardiac activity on transvaginal scan). In addition, reproductive outcomes; implantation rate, clinical pregnancy rate and miscarriage rate among obese and non-obese PCOS and non-PCOS patients were compared.

**Results:**

Our results revealed 38.5% clinical pregnancy rate in non-PCOs females, 23.8% in non-obese PCOS females whereas 26.4% in obese PCOS. Preclinical abortions were found to be highest (31.5%) in non-obese PCOS females and were the lowest (26.2%) in non-PCOS females. In non-PCOS group and non-obese PCOS females 35.4% and 44.6%, respectively, failed to become pregnant.

**Conclusion:**

The success after ICSI in terms of number of clinical pregnancies was more in non-PCOS patients as compared to PCOS. Increase in body mass index reflected a negative impact on the reproductive outcome in PCOS patients.

## Introduction

Polycystic ovary syndrome (PCOS) is found in 6-10% of the general population and considered to be a common hormonal disorder in females of reproductive age (1). According to the Rotterdam criteria, females suffering from PCOS have decreased or no ovulation, excessive androgen secretion and ultrasound evidence of poly cystic ovaries (2). More than 10 follicles, encircling an echo-dense stroma, measuring 2-8 mm in diameters, contained in an ovary sizing more than 10 CC in volume is diagnostic of PCOS on ultrasound. PCOS is manifested as changes in hormonal, reproductive and metabolic functions. Reproductive and hormonal disorders of PCOS include hypergonadism, anovulation, and increased secretion of Luteinizing hormone (LH) and early miscarriage risk. Metabolic disorders of PCOS include hyperinsulinemia, type 2 diabetes, obesity and hyperlipidemia. PCOS hormonal imbalances leading to anovulation is a significant cause of infertility (2). 

Primary infertility is found to be highly prevalent in South Asia, particularly in Pakistan with an estimated rate of 3% while secondary infertility in Pakistan is 18.4% Infertility, specifically associated with anovulation, is initially treated with anti-estrogen clomiphene citrate and exogenous gonadotropins. Unfortunately, this can increase the risk of multiple pregnancy rates, therefore many patients are given assisted reproductive treatment (ART) comprising of in vitro fertilization (IVF) and intra-cytoplasmic sperm injection (ICSI) (3). ICSI is a highly developed micromanipulation technique in which a single sperm is injected into an egg in vitro using micro-injectors (4). Obesity may establish a crucial barrier for effective fertility treatment in females who are PCOS. The relationship of PCOS and ICSI outcome has been the subject of observational studies. Obese females with PCOS had less number of retrieved oocytes and clinical pregnancy rates with a higher rate of abortions (5). In contrast, some studies suggested that obese and non-obese PCOS patients revealed no significant differences in IVF/ICSI results (6). 

This study aimed to assess the ICSI outcome in obese and non-obese women with PCOS by comparing the number of retrieved oocytes, implantation rate, clinical pregnancy rate and miscarriage rates. 

## Materials and methods

A cross sectional study was conducted from June 2015 till July 2016. The sample size was calculated in Open Epi software version 3 based on the formula; n= [DEFF*Np(1-p)]/ [(d2/Z21-α/2*(N-1) +p*(1-p)]. We required a sample size of 246 patients in order to achieve a confidence interval of 95% with a 20% prevalence of PCOS. Despite the sample size being 246, we surveyed 286 females to cover the drop-outs which can occur during the course of treatment. PCOS group was further classified into Non-Obese PCOS (body mass index (BMI <25 kg/m^2^, 130 females) and 91 females in Obese PCOS group (BMI ≥25 kg/m^2^). 

The clinical examinations monitored principles of Declaration of Helsinki. Females with primary infertility for at least for 2 yr, age range 20-40 yr and normal uterine cavity assessed on ultrasound, hysterosalpingogram or hysteroscopy were included. The first fresh IVF-embryo transfer cycle with long down regulation protocol was taken into consideration. Exclusion criteria consisted of females with known metabolic disorders (Diabetes, Hypertension, Metabolic Syndrome), pelvic pathologies (hydrosalpinges, myoma etc.), presence of stage IV (severe) endometriosis, and past history of myomectomy. All the cases that developed hyper stimulation were not included irrespective of the fact that cycle was canceled or not. PCOS was diagnosed by Rotterdam criteria; females who had decreased or no ovulation, excessive androgen secretion and ultrasound evidence of PCO were selected (2). 

The baseline investigations included measurement of BMI on the basis of South Asian Criteria (normal weight: 18.0-22.9 kg/m^2^, overweight: 23.0-24.9 kg/m^2^, obese: ≥25.0 kg/m^2^) (7); day 3 follicle stimulating hormone (FSH) and antral follicle count done by transvaginal scan (TVS). The treatment protocol of females from down regulation, controlled ovarian stimulation, ovulation induction (OI), oocyte pick up and embryo transfer was carried out as mentioned (8). The preovulatory follicle count (PFC) and endometrial thickness were measured on OI day. On the basis of beta-human chorionic gonadotropin (β-hCG) done 2 wk after egg collection and TVS performed after another 2 wk after identification of clinical pregnancy from pre-clinical abortion.

On the basis of β-hCG and TVS, results were categorized into non-pregnant with β-hCG <25 mIU/ml and preclinical abortion β-hCG >25 mIU/ml with no fetal cardiac activity on TVS. Clinical pregnancy was identified by the existence of a gestational sac with cardiac movement perceived by TVS. Oocyte recovery rate was number of oocytes obtained in comparison with visible follicles on TVS (9). Fertilization rate was determined by formation of pronuclei from the microinjected oocytes. The implantation rate was determined by appearance gestational sacs on TVS with respect to number of transferred embryos (10). A pregnancy rate was estimated by existence of an intrauterine gestational sac on TVS; per number of patients in the cycle (9).

**Ethical consideration**

Ethical approval was obtained from the Institutional Review Board of Islamabad Clinic Serving Infertile Couples (256-ICSFC-REC-16). Informed written consent was obtained from every participant. 

**Statistical analysis**

Computational data was stored and analyzed by employing SPSS software (Statistical Package for the Social Sciences, version 21.0, SPSS Inc., Chicago, IL, USA). Quantitative variables were computed for Mean±SD. Analysis of Variance (ANOVA) with Tukey’s HSD test was applied for post-hoc analysis, and significance at p-value of 0.05.

## Results

Out of an aggregate of 286 infertile females undergoing ICSI procedure included in data analysis, 65 (22.7%) were normal females (non-PCOS group) and 221 (77.3%) were PCOS females. Of the 221 PCOS females, 130 were included in non-obese PCOS group (BMI <25) and 91 females were included in obese PCOS group (BMI ≥25). Mean±SD of oocyte parameters of the 3 groups are summarized in Table I. The three groups did not differ with regards to age. PFC and number of oocytes/patient were found to be higher in obese PCOS group. The quality of embryos was graded as good (Grade I), fair (Grade II) and poor (Grade III) on the basis of cleavage rate and differentiation before embryo replacement. Good quality embryo with blastomeres of same size, slight or no fragmentation, and a zona pellucida that is not enormously dense in appearance; fair with blastomeres of same size, little fragments in cytoplasm wrapping <10% of embryo. Poor quality embryo had blastomeres of noticeably uneven size and moderate-to-significant fragments of cytoplasm, wrapping >10% of embryo external (11) 

The number of fertilized oocytes, number of cleaved embryos and grade 1 embryos were more in non-PCOS group as compared to non-PCOS group (Table I). Endometrial thickness did not differ widely among the three groups. Basal progesterone was significantly low (9.69±3.66 ng/ml) in non-obese PCOS females. Progesterone on OI day and estradiol on day of ovulation were found to be lower in non-obese PCOS females.

Table II compares the reproductive outcomes in the three groups. Fertilization rate and cleavage rate were the lowest in obese PCOS females and were the highest in non-PCOS females. Implantation rate was significantly high (62.31±32.31) in non-PCOS group compared to obese PCOS (26.37±38.01) and non-obese PCOS group (23.46±39.79).

Table III compares the pregnancy outcomes among the three groups. 38.5% of the non-PCOS females achieved clinical pregnancies whereas only 23.8% non-obese PCOS females achieved clinical pregnancies. Similarly, preclinical abortions were found to be highest (31.5%) in non-obese PCOS females and were the lowest (26.2%) in non-PCOS females. 44.6% non-obese PCOS females never got pregnant while only 35.4% non-PCOS females failed to get pregnant.

**Table I:** Comparison of characteristics of study groups

**Variables**	**PCOS (n= 221)**		**Non-PCOS (n=65)**	**p-value**
	**Obese (n=91)**	**Non-Obese ** **(n=** **130)**		
Female age (yr)	32.02 ± 4.81	32.42 ± 4.45	31.34 ± 4.63	0.302
Pre-ovulatory follicle count	10.86 ± 1.66	9.28 ± 1.57	8 ± 1.61*^	<0.001
No of oocytes/patient	9.86 ± 1.66	8.45 ± 1.71	7.86 ± 1.54*^	<0.001
No of oocytes Metaphase II	6.71 ± 1.09	7.07 ± 1.62	7.52 ± 1.64^	0.004
No of oocytes fertilized	4.35 ± 1.03	4.88 ± 1.33	6.03 ± 1.55*^	<0.001
No of cleaved embryos	3.89 ± 1.3	4.45 ± 1.5	5.68 ± 1.53*^	<0.001
Grade_1	0.99 ± 0.1	1.59 ± 0.49	2.71 ± 0.76*^	<0.001
Grade_2	1.9 ± 0.34	1.95 ± 0.57	1.68 ± 0.56*^	0.001
Grade_3	1.65 ± 0.77	1.32 ± 0.47	0.74 ± 0.54*^	<0.001
Endometrial thickness (mm)	8.8 ± 3.2	8.05 ± 3.24	8.14 ± 3.63	0.231
Basal progesterone (ng/ml)	14.17 ± 7.65	9.69 ± 3.66	14.3 ± 9.39*	<0.001
Progesterone on ovulation induction day (ng/ml)	1.64 ± 0.74	1.46 ± 0.76	1.55 ± 0.68*^	0.220
Progesterone on embryo transfer day (ng/ml)	146.43 ± 63.51	154.37 ± 58.95	137.62±64.11*^	0.195
Estradiol on ovulation induction day (pg/ml)	2342.71±243.0	2139.43±411.7	2267.2±243.6^	<0.001
Estradiol on embryo transfer day (pg/ml)	891.27±119.39	856.07±173.86	881 ± 115.54^	0.188

Data presents values as Mean±SD.

ANOVA test was applied to calculate difference between groups and p <0.001 was considered significant.

PCOS: Polycystic ovary syndrome

* p value <0.05, non PCOS with non-obese PCOS

^ p value <0.05, non PCOS with obese PCOS

**Table II:** Comparison of reproductive outcome in PCOS and non-PCOS

**Rates**	**PCOS (n= 221)**		**Non PCOS (n=65)**	**p-value**
	**Obese (n=91)**	**Non-Obese (n=130)**		
Oocytes retrieval rate	90.58 ± 1.38	90.7 ± 4.43	98.54 ± 4.78*^	<0.001
Oocyte maturity rate	69.059	83.800	95.504*^	<0.001
Fertilization rate	64.64 ± 10.05	68.17 ± 5.62	79.61 ± 10.59*^	<0.001
Cleavage rate	87.67 ± 16.55	91.26 ± 17.72	94.42 ± 7.68*^	0.028
Implantation rate	26.37 ± 38.01	23.46 ± 39.79	62.31 ± 32.31*^	<0.001

Data presents values as frequencies in percentages using the following formulas.

Oocyte retrieval rate= Number of Oocytes/ PFC ×100 (PFC: preovulatory follicle count)

Oocyte Maturity rate= Number of mature oocytes=Mature oocytes/ number of oocytes ×100

Fertilization rate= Pronuclei/ mature oocytes ×100

Cleavage rate= Cleaved embryo/ fertilized embryo ×100

Impanation rate= Number of sacs/ number of embryos transferred×100

* p value <0.05, non PCOS with non-obese PCOS

^ p value <0.05, non PCOS with obese PCOS

**Table III:** Outcome after ICSI in study Groups

**Pregnancy**	**PCOS (n= 221)**		**Non-PCOS (n= 65)**
	**Non-Obese (n= 130)**	**Obese (n= 91)**	
Not pregnant	42.9	44.6	35.4*^
Preclinical abortion	30.8	31.5	26.2*^
Clinical pregnancies	26.4	23.8	38.5*^

Data presented as percentage.

PCOS: Polycystic ovary syndrome

* p value <0.05, non PCOS with non-obese PCOS

^ p value <0.05, non PCOS with obese PCOS

## Discussion

Results of current research revealed that clinical pregnancies rate was highest in non-PCOS females followed by non-obese PCOS and then obese PCOS. Similarly, preclinical abortions were found to be maximum in non-obese PCOS females and lowest in non-PCOS females. Around 44.6% non-obese PCOS females never got pregnant while only 35.4 % non-PCOS females failed to get pregnant in the study. Although the effect of BMI on IVF/ICSI outcomes remain controversial due to different inclusion criteria and the cutoff point of BMI yet we discovered that increase in BMI effected the pregnancy outcome in PCOS patients. 

PCOS females experiencing fertility management have a much higher possibility of multiple pregnancies and other adverse complications as a result of ovarian hyperstimulation. Due to the failure of less invasive techniques, IVF/ICSI is deliberated as best treatment choice intended to restore fertility whereas minimizing the chance of multiple gestations. The association of obesity with PCOs has been observed in 30-75% of females (12). Recent data proposes that, irrespective of PCOS, pregnancy attainment and preservation is poorly pretentious by an increase in BMI. Obese women thus experience lesser options of clinical pregnancy with an increased miscarriage rate after getting infertility management. In our study, we found that PFC was higher in PCOS groups and lower in non-PCOS group. This is attributed to hormonal disturbances in PCOS females resulting in the development of multiple small follicles that do not ovulate each month. Others have previously reported similar results (13). 

Ciepiela and coworkers also found that the number of follicles, as well as oocytes, was higher in PCOS group even at a lower dose of gonadotropins (14). Number of oocyte per patient was found to be higher in PCOS groups and lower in non-PCOS group confirming previous studies. It has been observed that obesity, as well as presence of PCOS, results in reduction in size of oocytes (15, 16). Increased number of MII oocytes is associated with positive ICSI outcomes (8). MII oocytes were found to be the highest in non-PCOS group in our study. Similar results were found in previous studies (15).

Some studies have shown higher number of MII oocytes in PCOS females but this was mainly due to the fact that PCOS females develop greater number of oocytes. In these studies, percentage of MII oocytes was still higher in non-PCOS females (14). Our results showed a negative association is existed between MII oocytes with BMI supported by others (13). Number of fertilized oocytes was found to be lowest in obese PCOS group and lower in non-Obese PCOS group. In contrast, other studies showed increased number of oocytes were fertilized in PCOS females (14). However, Hind and colleagues proposed that BMI and PCOS have no significant effect on the number of oocytes fertilized (17). There is an increased incidence of pregnancy loss in PCOS as a result of the inborn endocrine disruption linked to the syndrome (18). 

Our results revealed that rate of clinical pregnancy in non-PCOS females was 38.5% whereas it was 23.8% in non-Obese PCOS females. Similarly, preclinical abortions were found to be highest (31.5%) in Obese PCOS females and were the lowest (26.2%) in non-PCOS females. Our data revealed that the clinical pregnancy rate in non-PCOS females was higher whereas failure of pregnancy was highest in Obese PCOS females. The negative impact of increase in weight on the quality of oocyte without PCOS has been documented by a decrease in number of transferable embryos in obese, females. A subgroup enquiry discovered smaller oocytes in the obese control patients compared to the non-obese control infertile patients proposing that obesity affects oocyte size independent of PCOS. (15) Our results demonstrated that preclinical abortions were found to be highest (31.5%) in obese PCOS females and were the lowest (26.2%) in non-PCOS females. Some scientists concluded in their study that, the presence of PCOS in non-obese patient may be a favorable prognostic factor before considering IVF (14). 

Wang and colleagues emphasized that in young PCOS females (less than 25 yr of age) BMI should be reduced to 26 kg/m2 before initiation of ART (16, 19). Overweight and obese PCOS patients should thus be encouraged to lose weight before initiation of ART (6). It was observed that BMI impaired endometrial receptivity in assisted reproductive treatment protocols (16, 20) Higher implantation and pregnancy rates were acquired with an endometrial thickness of 7-8 mm in our population (21). However, we did not observe any significant difference in endometrial thickness in PCOS and non-PCOS to impact on pregnancy outcome.

Okohue observed likelihood of becoming pregnant with endometrial thickness 8-14 mm (22). The number of clinical pregnancies acquired after ICSI was high in non-PCOS patients as compared to PCOS. Increase in BMI of PCOS exerted a dual effect, decreased the number of clinical pregnancies and enhanced the number of non-conceptions and pre-clinical abortions. Our study did not exclude the females who had infertility because of male factor, since we wanted to compare results of ICSI in females with and without PCOS, hence impact of severe male factor on IVF outcome could not be excluded which is a limitation. Thus it is recommended to induce weight reduction strategies for PCOS patients before initiation of ART for enhancing the results of IVF/ICSI.

The success after ICSI in terms of number of clinical pregnancies was more in non-PCOS patients as compared to PCOS. Increase in body mass index reflected a negative impact on the reproductive outcome in PCOS patients. Since PCOS is mostly occurred with obesity, it is essential to decrease weight in PCOS patients for attainment of improved outcomes of IVF/ICSI.
